# Secondary Metabolism Rearrangements in *Linum usitatissimum* L. after Biostimulation of Roots with COS Oligosaccharides from Fungal Cell Wall

**DOI:** 10.3390/molecules27072372

**Published:** 2022-04-06

**Authors:** Redouan Elboutachfaiti, Roland Molinié, David Mathiron, Yannis Maillot, Jean-Xavier Fontaine, Serge Pilard, Anthony Quéro, Clément Brasselet, Marguerite Dols-Lafargue, Cédric Delattre, Emmanuel Petit

**Affiliations:** 1 UMRT INRAE 1158 BioEcoAgro, BIOlogie des Plantes et Innovation (BIOPI), Université de Picardie Jules Verne, IUT d’Amiens, Avenue des Facultés, Le Bailly, 80025 Amiens, France; roland.molinie@u-picardie.fr (R.M.); yannis.maillot@etud.u-picardie.fr (Y.M.); jean-xavier.fontaine@u-picardie.fr (J.-X.F.); anthony.quero@u-picardie.fr (A.Q.); emmanuel.petit@u-picardie.fr (E.P.); 2 Plate-Forme Analytique, Université de Picardie Jules Verne, 33 Rue Saint Leu, 80039 Amiens, France; david.mathiron@u-picardie.fr (D.M.); serge.pilard@u-picardie.fr (S.P.); 3 Université Clermont Auvergne, Clermont Auvergne INP, CNRS, Institut Pascal, 63000 Clermont-Ferrand, France; clement.brasselet@uca.fr (C.B.); cedric.delattre@uca.fr (C.D.); 4 EA 4577 Œnologie, INRA, USC 1366, ISVV, Bordeaux INP, Université de Bordeaux, 30072 Bordeaux, France; marguerite.dols@enscbp.fr; 5 Institut Universitaire de France (IUF), 1 Rue Descartes, 75005 Paris, France

**Keywords:** COS oligosaccharides, *Linum usitatissimum* L., secondary metabolites, seedlings, roots elicitation

## Abstract

In vitro culture of flax (*Linum usitatissimum* L.) was exposed to chitosan oligosaccharides (COS) in order to investigate the effects on the growth and secondary metabolites content in roots and shoots. COS are fragments of chitosan released from the fungal cell wall during plant–pathogen interactions. They can be perceived by the plant as pathogen-associated signals, mediating local and systemic innate immune responses. In the present study, we report a novel COS oligosaccharide fraction with a degree of polymerization (DP) range of 2–10, which was produced from fungal chitosan by a thermal degradation method and purified by an alcohol-precipitation process. COS was dissolved in hydroponic medium at two different concentrations (250 and 500 mg/L) and applied to the roots of growing flax seedlings. Our observations indicated that the growth of roots and shoots decreased markedly in COS-treated flax seedlings compared to the control. In addition, the results of a metabolomics analysis showed that COS treatment induced the accumulation of (neo)lignans locally at roots, flavones luteolin *C*-glycosides, and chlorogenic acid in systemic responses in the shoots of flax seedlings. These phenolic compounds have been previously reported to exhibit a strong antioxidant and antimicrobial activities. COS oligosaccharides, under the conditions applied in this study (high dose treatment with a much longer exposure time), can be used to indirectly trigger metabolic response modifications *in planta*, especially secondary metabolism, because during fungal pathogen attack, COS oligosaccharides are among the signals exchanged between the pathogen and host plant.

## 1. Introduction

Oilseed flax is grown primarily for its seed, unlike textile flax, which is used in the textile industry and for the development of composite materials [1]. *Linum usitatissimum* L. (linseed and fiber flax cultivars) is one of the oldest domesticated plants; its use dates back to about 9000–8000 BC [2]. The Latin meaning of the name *usitatissimum* (“the most useful”) highlights the importance of this plant. In Europe, flax and linseed are terms used to refer to this crop, describing seeds grown for fiber and those produced for oil, respectively [3]. Flax use was particularly popular in ancient Egypt in the time of the Pharaohs, where flax oils were used to embalm the dead and linen was used to wrap mummies [4]. Linseed was used in the preparation of bread [5] or for its laxative properties [6].

According to the FAO statistics (food and Agriculture Organization of the United Nations), global linseed production has fluctuated considerably in recent years, after declining during the period 1971–2020 and ending at 3.37 million tons in 2020. Farmers have moved away cultivating flaxseed, which offered lower yields than most other cereals (e.g., wheat and corn) or oilseeds (e.g., rapeseed) [1]. Concomitantly, from 2015 to 2020, the flaxseed market grew by 4.2% in value. Flaxseed sales were high in the industrial sector, accounting for nearly 20% of the market share; this was also supported by use in different industries such as food, nutraceuticals, animal feed [7,8]. Thus, the production of flaxseed is expected increase in the coming years.

The specific attributes of flaxseed oil come from its fatty acid profile, dominated by the presence of alpha-linolenic acid (C18:3 belonging to the omega 3 family), comprising more than 54% of the total on average. In Europe in the 20th century, the use of its oil increased significantly in nonfood sectors [9]. Thus, thanks to its excellent drying properties and reactivity, flaxseed oil has proven to be the raw material of choice for the manufacture of linoleum (floor covering), paints and inks, as well as soaps [10,11,12]. At the same time, cakes, i.e., coproducts of the oil during the crushing of the seed, have been successfully used in animal feed, particularly with cattle breeders, probably due to their high residual fat content and the presence of omega 3, the benefits of which were not explicitly known previously [13].

Flax is an environmentally friendly crop requiring little maintenance, and is also a significant asset in crop rotation [14]. Like any large crop, it is subject to the impact of pathogen attacks, which can lead to decreases in production yield [15]. One of the most common pathogens is *Fusarium*, which is estimated to be responsible for more than 20% of the damage associated with flax cultivation [16]. This type of fungus, called necrotrophic, secretes enzymes, such as polygalacturonases, which allow them to enter the host plant by degrading the plant cell wall. The production of a large quantity of degrading cell wall enzymes (cellulases, pectinases, proteases, endo-xylanases, endo and exo-polygalacturonases, etc.) facilitates the colonization of the host plant [17].

In order to defend itself, the plant must detect the attack by the pathogen; this depends on signals or molecules. Numerous studies have focused on molecules whose origins can be exogenous (Pathogen Associated Molecular Pattern (PAMP), originating from the pathogenic agent) or endogenous (molecules originating from the plant itself, i.e., Damage Associated Molecular Pattern (DAMP), resulting from the action of pathogens). The scientific community has been developing original and environmentally friendly strategies for many years to fight against such pathogens. One approach is based on the use of natural or non-natural compounds, called elicitors, which make it possible to induce an immune response in the plant in order to protect it against attacks by pathogens. Plants respond to attacks by pathogens or elicitor treatments by activating a wide variety of protective mechanisms aimed at preventing the replication of the pathogen [18]. Defense mechanisms include the production of reactive oxygen species (ROS), the activation of defense genes, the synthesis of defense proteins, cell wall strengthening, and the accumulation of secondary metabolites. These stress-induced secondary metabolites are collectively called phytoalexins and are considered to be molecular markers of disease resistance. Among these secondary metabolites, the activity of polyphenols in plant disease resistance relies to a large extent on their cytotoxicity [19]. Moreover, these antioxidant phenolic compounds also seem to play an essential role in counteracting the effect of one of the responses activated just a few minutes after the attack of the pathogen, i.e., the production of ROS which damages the plant itself [20]. These compounds, isolated in most plant organs (including stems, leaves, roots and fruits or seeds), differ depending on the plant and their location. Many of these compounds are synthesized in the phenylpropanoid pathway and give rise to a wide variety of secondary metabolites such as flavonoids, catecholamines, phenylpropanoic acids, phenols, lignins and tannins. Concerning phenylpropanoic acids, for some of them, the increase, of their biosynthesis is closely linked to the components of the cell wall (lignification). Flavonoids perform various biological activities in plants, like protecting against insect and herbivores, regulating interactions with symbiotic microorganisms (soil fungi or bacterial), allelopathic plant–plant interactions, direct or indirect action against pathogenic fungi or bacteria, and, according to the properties of the scavenging free radicals species, defense against abiotic stress like UV radiation and temperature [21].

In flax, the flavonoids identified to date largely comprise flavone *C*-glycosides. Although their biological roles have not yet been comprehensively described, they are known to exert antifungal activities against the pathogen *Fusarium oxysporum* [16]. Two of these compounds, i.e., swertisin and swertiajaponin, are putatively involved in cold tolerance mechanisms in response to cold stress in winter flax [22].

It has notably been shown that plant metabolisms, and consequently, the production of secondary compounds, can be affected by the application of a wide range of natural saccharide elicitors. Many of these bioelicitors have been isolated from algae (ulvans, carrageenans, fucoidans, alginates, etc.) [23,24], plants (galacturonans, etc.) [25] and other organisms (chitin, rhamnolipids, etc.) [26,27]. To date, some of these have been marketed and approved as elicitors, like laminarin (Iodus 40, Goëmar), chitosan (Elexa, Glycogenesis), or more recently, an elicitor based on oligochitosan and oligoglacturonans (Fytosave, Fytofend).

Laminarin is a *β*-(1,3)-glucan extracted from a brown alga (*Laminiaria digitata*) whose chemical structure is close to that of fungal glucan elicitors. The first responses of the plant consist of calcium fluxes, an oxidative “burst” and an alkalinization of the extracellular medium. Then, laminarin induces the expression of defense genes associated with phenylpropanoid pathways, and defense proteins (in particular *β*-(1,3)-glucanase). Furthermore, studies on sulfated laminarin have shown that this chemical modification can induce more effective protection than native laminarin against the tobacco mosaic virus [28].

Fragments of chitin or chitosan from the exoskeletons of crustaceans (shrimps crabs) or from the cell walls of fungi have also been widely studied. The chemical structure consists of N-acetyl D-glucosamine units linked by β-(1,4) bonds which are totally or partially acetylated. The size and degree of acetylation of chitosan oligomers plays an important role in resistance induction. Moreover these compounds can act directly on the growth of pathogens (antibacterial or antifungal activities); however, it has also been reported that they can modulate the growth of the exposure plant (i.e., acting as biostimulants) [29].

The pectic fragments of the plant wall have been evidenced as the first elicitors. These fragments, essentially generated by the action of fungal endopolygalacturonases, induce a cascade of events leading to the expression of resistance. In order to be active, the oligogalacturonans (OGAs), formed by the enzymatic cleavage of long chains of homogalacturonans, must be of a certain size which seems to depend on the plant itself. However, OGAs with a degree of polymerization (DP) lower than 5 or higher than 18 have little or no eliciting activity [25].

In the present study, we used a hydroponic system to examine the capacity of COS oligosaccharides, obtained from fungal cell walls, to induce morphological changes and evaluate the metabolomic responses in the roots and shoots of flax seedlings. Several studies have shown the importance of root treatments to evaluate effects of COS oligosaccharides on different plant species [30,31,32,33,34]. This practice was chosen because the roots play numerous important roles in plant growth and development processes [34,35]. COS oligosaccharides are often associated with the production of growth regulators that can control or modify various mechanisms in the roots as well as the aerial part of the plant.

Hydroponic cultures were carried out in Hoagland medium, and two concentrations of COS were applied to the roots. The first aim of this study was to develop a process to produce a fraction of purified COS oligosaccharides containing lower-DP obtained from fungal chitin under autoclaving conditions. The second aim was to establish a standardized development protocol with which to evaluate the effect of these oligosaccharides on flax seedlings.

## 2. Results

### 2.1. Preparation of Chitosan Oligosaccharides Elicitors

#### 2.1.1. Chitosan Degradation and Chitosan Oligosaccharides Preparation

The fungal chitosan fraction (F1) was provided by Biolaffort (Floirac, France) and was used without any further purification steps. The average molecular weight (Mw) was evaluated to be 32 kDa using HPLC/SEC analysis, and the degree of acetylation (DA) was estimated to be 9.6% by ^1^H NMR analysis (data not shown) [36,37,38]. Many studies indicate that the degree of deacetylation depends on the extraction method and treatment conditions used for the preparation of commercial fungal chitosan [39,40,41,42]. 

Acidic hydrolysis of chitosan has been widely investigated in recent years [43,44,45,46]. Acid solutions are typically hydrochloric acid or acetic acid, as are well-documented in the literature and commonly used in various industrial processes [47,48,49]. 

**Figure 1:**
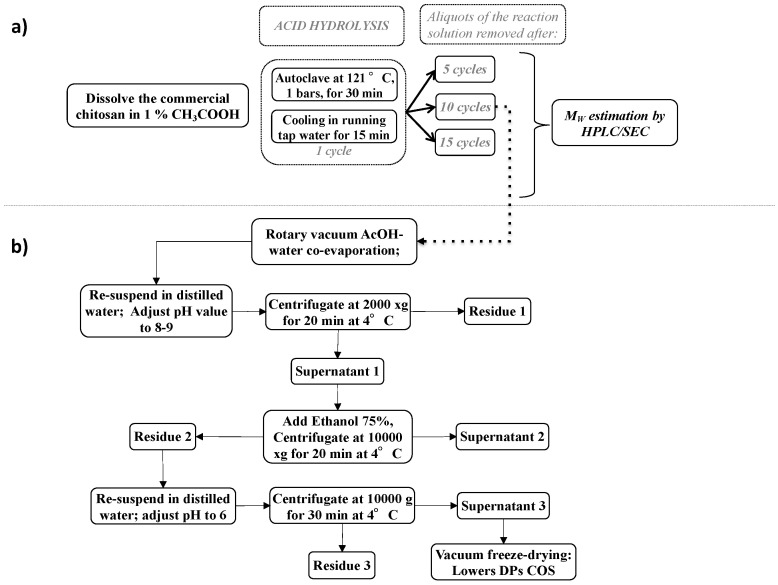
Schematic diagram of fungal chitosan acid hydrolysis (**a**) and chitosan oligomer production (**b**).

Generally, high concentrations of hydrochloric acid (0.5–2 M) and higher temperatures (100–121 °C) associated with high pressure (1–2 bars) are most commonly used to increase the levels of chitosan depolymerization. In the present study (Figure 1a), the fungal chitosan (F1) was dissolved at 2% (*w*/*v*) in 1% (*v*/*v*) acetic acid and subjected to 5 to 15 autoclaving/cooling cycles of 30 min each at 121 °C (1 bar) with a 15 min temperature ramp-up and a 15 min ramp-down for each thermal cycle, according to a modified protocol of No et al. [44]. To determine the M_w_ distribution of F1 after thermal treatment, aliquots from each experimental conditions were collected, and the resulting products of degradation were characterized using high-performance liquid chromatography size exclusion chromatography (HPLC/SEC) [38].

**Figure 2:**
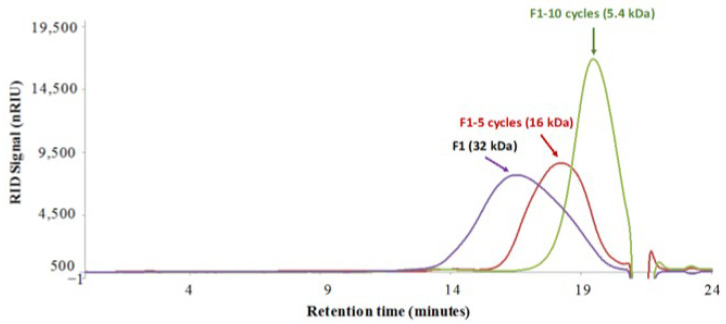
HPLC/SEC analysis (on TSK Gel 5000/3000 PWXL placed in serial) of depolymerized fungal chitosan (fraction F1) via an acetic acid/autoclaving process. The average molecular weight was determined using a calibration curve of pullulan standards.

The impact of thermal acid hydrolysis on the molecular weight of chitosan appears to be dependent on the number of autoclaving/cooling cycles (Figure 2). Maximum depolymerization was investigated with up to 10 cycles (121 °C, 1 bars, 30 min) in order to obtain the lower DP oligomer fractions, after which a decrease in the M_w_ and final product quality were appreciable for this study. As observed in Figure 2, the chitosan oligomers obtained with 10 autoclaving cycles had a homogenous average Mw of 5.4 kDa, constituting a good average M_w_ target before investigation of their elicitation effect on flax seedlings [29]. According to the literature [44,50], the percentage of M_w_ decrease was estimated to be 83.1%, calculated as follows: MW decrease (%) = (MWi − MWf)/MWi × 100, where MWi is the initial molecular weight and MWf is the molecular weight after hydrolysis.

The chitosan oligomer fraction with an average Mw of 5.4 kDa was subjected to a further purification step. After cooling following the last cycle, excess acetic acid was removed by coevaporation with water at reduced pressure. The highest DP in the hydrolysis solution was eliminated because it was considered to be less suitable for the optimal elicitation strategy based on the application of chitosan oligomers to plant roots [29]. The highest DP oligomers (Residue 1, Figure 1b) were precipitated by increasing the pH of the hydrolysate to 8.5 with the addition of concentrated NaOH (1 M). The lowest DPs (Residue 2, Figure 1b) were recovered from the reaction mixture by using specific precipitation with seven volumes of ethanol, and centrifugation. The obtained material was dissolved in distilled water and centrifuged, and the supernatant was freeze-dried to generate a brownish powder with low DP chitosan oligomer. It should be noted that the yield of this fraction was estimated to be 21% by weight of the starting material (F1 fungal chitosan fraction).

#### 2.1.2. Structural Characterization of Chitosan Oligomers by ESI-HRMS and NMR Analysis

In order to obtain more accurate data on the Mw of the generated chitosan oligomers, mass spectrometry analyses were undertaken using direct injection electrospray ionization-high resolution mass spectrometry (ESI-HRMS) to estimate the DP distribution.

**Figure 3:**
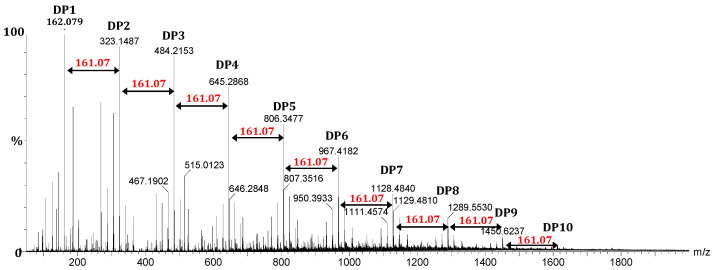
Electrospray-ionization high resolution mass spectrometry (ESI-HRMS) analysis of chitosan oligosaccharides obtained from fungal chitosan by an acetic acid hydrolyzed method using an autoclaving-cooling process (10 cycles, 30 min at 121 °C and 15 min cooling).

Figure 3 is a representative ESI-HRMS spectrum of chitosan oligomers produced during the acid hydrolysis process. The analysis was performed in positive ion mode, showing mainly the [M+H-H_2_O]^+^ions of the different oligomers (from DP1 to DP10) obtained following in-source spontaneous water loss. The mass difference of 161.07 Da between the two consecutive DP ions corresponded to the residual mass of the repeating glucosamine unit (C_6_H_11_O_4_N), clearly identifying chitosan oligosaccharides (COS). The spectrum also reveals that the chitosan oligomers fraction was mainly composed of fully deacetylated COS with DPs from 2 to 10. The mass of the major singly charged molecular ion was calculated to be [161.07n + [1.00 (H) + 17.00 (OH)] (mass of reducing end residue) + 1.00 (H)]−M_H2O_ Da respectively, where n is the number of GlcN units (corresponding also to DP, as reported in Supplementary Data Table S1). Table S1 (*m*/*z* of the COS ion) was created based on unacetylated COS, corresponding to the major species. The presence of COS containing acetyl groups is illustrated for the DP3 mass spectrum region in Supplementary Figure S1. Indeed, the ion at *m*/*z* 544.2352 and 526.2244 match the [M+H]^+^ and [M+H-H_2_O]^+^ ions of a DP3 bearing one acetyl group.

The DP distribution of COS was evaluated by a method of UPLC-SEC/ESI-HRMS developed in the laboratory [51]. It displayed a bimodal-distribution of the molecular weight profiles with one peak centred at DP 6–7. As expected, the N-acetyl glucosamine residue (GlcNAc) was present at relatively low concentrations, since the F1 fraction possessed a DA < 10%. Similar observations have been reported previously [52]. 

**Figure 4:**
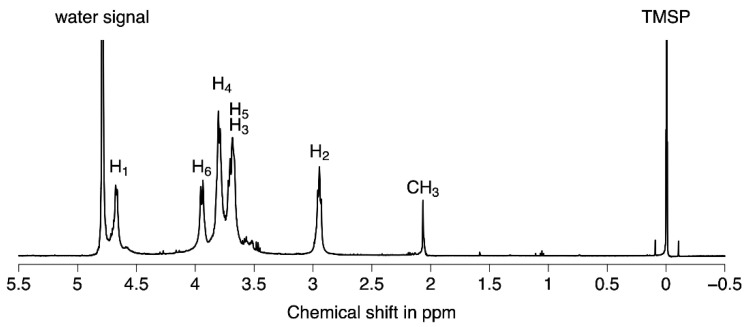
^1^H NMR spectra (600 MHz, in D_2_O) of COS oligosaccharides. The assignments of peaks were deduced from the literature [53].

The NMR (1D ^1^H) spectra (Figure 4) were recorded in D_2_O at 25 °C on a Bruker Avance III 600 spectrometer (with TMSP as the internal standard). The assignments of the proton signals were made based on coupling patterns and double resonance experiments, and the values of chemical shifts were deduced from data for GlcN and GlcNAc oligomers published in the literature [53]. 

The ^1^H-NMR spectrum of COS (Figure 4) showed a doublet peak at *δ* 4.69 ppm that could be attributed to the anomeric hydrogen (H1) of the GlcN unit connected to anomeric carbon (C1), which indicated that the saccharide residues in COS had β configurations. The signal around *δ* 3.00 ppm can be assigned to H2 from the GlcN unit (C2). Several signals were observed in the range of *δ* 3.7 to *δ* 4.0 ppm which corresponded to nonanomeric hydrogens H3–H6 in the sugar ring. The minor peak at *δ* 2.07 ppm is characteristic from CH3 protons of acetyl groups, revealing the presence of few residual N-acetyl groups in the structure of the COS oligosaccharides (around 6%). It should be noted that the NMR ^13^C spectrum of COS (Supplementary Table S2) showed the characteristic ^13^C signals of chitosan patterns [53].

These results clearly indicate the presence of pure lower DP of COS oligosaccharides (DP 2 to 10, DA = 6%) before elicitation assay investigations of flax seedlings.

### 2.2. Defense Responses of Flax Seedlings Activated by Chitosan Oligosaccharides (COS) Elicitation

#### 2.2.1. Phenotypic Observations

In our experiment, the effect elicitors of COS (DP 2–10, DA = 6%) were evaluated on four-week-old flax seedlings, variety “Marquise”, originating from seed germination in spring. Flax seedlings were cultivated on Hoagland medium supplemented with two different concentrations of COS (250 and 500 mg/L) and compared with control (Hoagland medium without COS). COS in the roots of flax seedlings were perceived as signals of putative fungal pathogen attacks [54], as judged by the presence of visible symptoms such as typical, brownish lesions on lateral root tips detected 24 h after contact with COS oligosaccharides. These lesions became enlarged and finally spread throughout the entire crown and main root, causing tissue maceration.

On other hand, the aerial parts of the seedlings were characterized by a little wilting and yellowing symptoms beginning at shoots tips 48 h after root COS treatment and progressing rapidly to the underlying young leaves. One-week post-treatment, the aerial parts of the treated seedlings showed clear sign of stress. 

Although the mechanism underlying this observation is still unknown, it is possible that the application of exogenous COS oligosaccharides influenced the concentration of plant defense compounds, such as flavonoids or phenolic acids in the above- and below-ground tissues [55]. COS oligosaccharides are known to induce the production of phenolic compounds and specific phytoalexins with antifungal activity [56,57]. Biosynthesis is known to occur not only in the feeding sites of treated seedlings, but also in systemic roots and aerial tissues. In addition, COS induces the biosynthesis of lignin-based phenolic monomers which polymerize in cell walls as a physical barrier against pathogens [58]. 

#### 2.2.2. UPLC-MS Data and Metabolite Identification

In order to gain insights into the metabolic variation caused by COS oligosaccharide treatment, tissues (roots and shoots) were chosen based on visual exploration of plant physiology and the first appearance of symptoms. Root tissues are appropriate to identify responses directly regulated by COS oligosaccharides, making it possible to explore the putative systemic reprogramming of metabolic pathways as a possible coordinated and integrative response of flax seedlings to COS biostimulation. Consequently, the possible root local metabolic changes during COS biostimulation, associated with systemic effects in the shoots, can provide a broader perspective on plant protection in the context of fungus–flax interactions. 

Polar extracts from shoots and root tissues were performed at various sampling times for the control condition and COS-treated seedlings. Samples preparations for metabolomics analyses were subjected to an UPLC-MS system with an electrospray ionization interface. Based on the nontargeted metabolomics method, the AMOPLS discriminant analysis score plot was investigated to determine similarities and differences between control and treated samples. 

A data matrix consisting of feature intensities associated with *m*/*z* values and retention time for the samples was imported into the Matlab software (The MathWorks, Natick, MA, USA) for multivariate AMOPLS analysis. The AMOPLS analysis considers the full factorial design in the context of exploratory data analyses with a simplified interpretation of signal variations related to the main effects or interactions between experimental factors. 

Using a cut-off of 200 and 100 for the average signal intensities of variables in shoots and roots respectively, 64 variables were obtained by this algorithm in shoot tissues for a total of 20 annotates, and 32 in root tissues for a total of 15 annotates. These data were scaled using a UV scale. 

Some compounds were identified in previous works [16,22,59], while others were annotated with exact masses and MS/MS. The identity of some metabolites was confirmed using authentic standards. Some of these partially annotated metabolites were classified as putative MS/MS annotations, while others were labeled unknown compounds. As presented in Table 1, different classes of metabolites were presented as cyanogenic glycoside (linamarin (1) and lotaustralin (2)), flavonoids (isoorientin (3), lucenin-2 (4), carlinoside (5), isovitexin (7), vitexin (8), vicenin-1 (9), vicenin-2 (10), triticuside A (11), schaftoside (12)), hydroxycinnamic acids (coniferin (14), HHMPG (15), 3-*O*-caffeoylquinic acid (17), caftaric acid (19), icariside F2 (20) and chicoric acid (21)) and (neo)lignans (SMG (31), PMG (23), PDG (24), LMG (25), DCG (24) and (-)-Olivil 4′-*O*-beta-D-glucopyranoside (30)). Nine variables seemed to be isomers of known metabolites. The MS/MS fragmentation pattern was compared with bibliographic data, as summarized in supplementary data Tables S3 and S4.

#### 2.2.3. Metabolic Changes in Flax Seedling Root and Shoot Tissues in Response to COS Applied Locally at Roots

**Figure 5:**
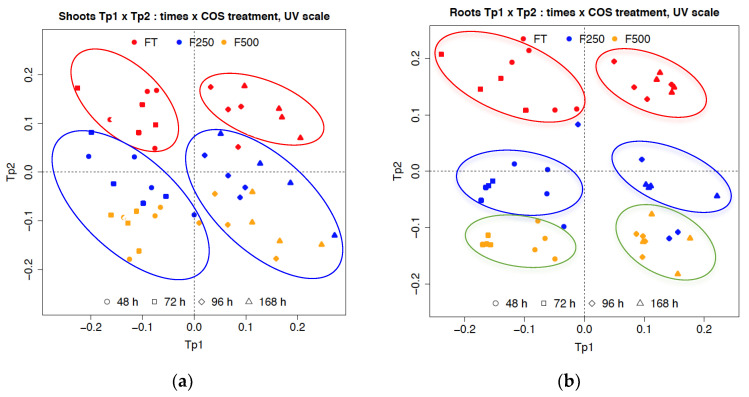
AMOPLS score plot: (**a**) shoots; (**b**) roots of flax seedlings from control (FT in red) and treated with COS solution (250 mg/L in blue and 500 mg/L in yellow) at four-time kinetic points (-◯- 48 h, -☐- 72 h, -◇- 96 h and -△- 168 h).

Figure 5a,b represent the AMOPLS score plots for data acquired in MS negative ion mode corresponding to the UPLC retention time range between 0.5–5.5 min. The score of the first predictive component was associated with the time effect (Tp1), whereas the second predictive component (Tp2) was linked to response to treatment with COS oligosaccharides. Considering these two principal components, we explained the variance in shoots as follows: COS concentration treatment 13%, time 31% and interaction COS concentration treatment–time 15% (Figure 5a). Similar results were obtained for roots samples: COS concentration treatment 16%, time 31% and interaction COS concentration treatment–time 20% (Figure 5b). 

Shoot samples were clustered into four groups (Figure 5a) represented by the control (FT) points (group 1: 48–72 h; group 2: 96–168 h) and COS treatment (F250, F500) points (group 3: 48–72 h; group 4: 96–168 h), assuming that each group could generate a distinct metabolite profile and time-related variation. The profiles obviously differed between the controls and groups treated with COS, whereas the profiles of samples treated with first COS concentration (250 mg/L) and those treated with second (500 mg/L) were located close together and could not be separated.

By comparison, for root samples (Figure 5b), two groups (48–72 h and 96–168 h) of treated samples with first COS concentration (250 mg/L) were separated from the samples treated with second COS concentration (500 mg/L). These results indicated that treatment of the roots of flax seedlings with first or second COS concentrations induced a marked metabolic difference 48 h after treatment. Moreover, the most prominent metabolic profile modifications were observed in root seedlings at 96 h and 168 h after treatment, compared to the controls.

**Figure 6:**
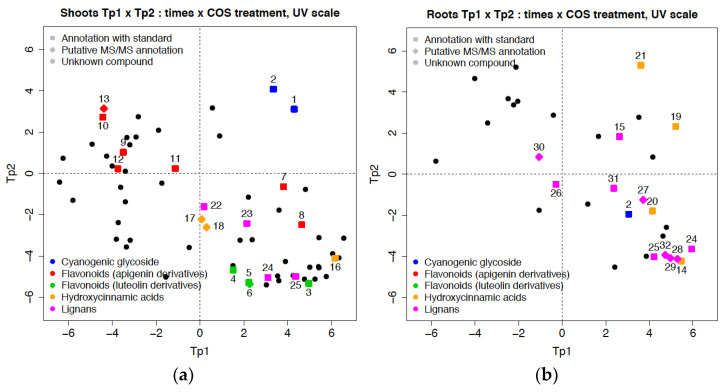
Loading plot of: (**a**) shoot metabolites; and (**b**) root metabolites of flax seedlings from the control and treated with COS solution.

The distinction depicted in the Tp1 and Tp2 parameters resulted from the changes in metabolite levels in the samples (roots and shoots) of controls and COS treated flax seedlings. As observed in Figure 6a, the contribution of metabolites to Tp1 discrimination in shoots was dominated by flavonoid apigenin derivatives (schaftoside isomer (6), vicenin-2 (10)) and cyanogenic glycosides (lotaustralin (2) and linamarin (1)); while other flavonoid luteolin derivatives (carlinoside (5), carlinoside isomer (6), orientin and iso-orientin (3)), (neo)lignans (PMG (23), DCG (24), LMG (25)) and hydroxycinnamics acids (chlorogenic acid (16)) were the major metabolite contributors to Tp2.

In the same way, as shown in Figure 6b, the contribution of metabolites in roots for tp1 was attributed to several compounds, notably chicoric acid (21), caftaric acid (19) and (neo)lignans (HHMPG (15)). The most discriminant metabolites contributing to the second orthogonal predictive component (tp2) were (neo)lignans (SMG isomer (32), DCG (24), olivil isomer 2 (28) and 3 (29)), coniferin (14), and lotaustralin (2).

**Figure A1:**
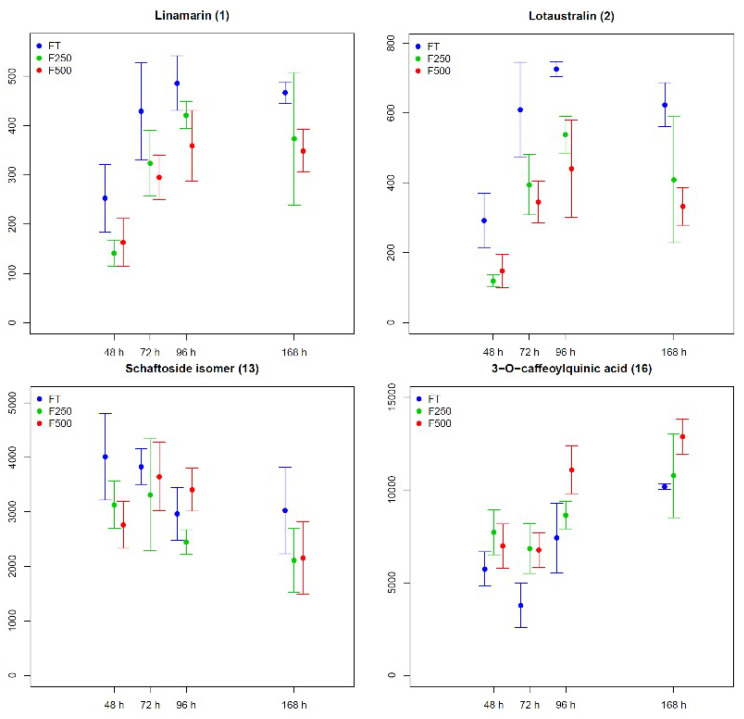
Comparison of some discriminating metabolite content in flax shoots from control (FT) and seedling treated with COS oligosaccharides solutions (F250 and F500).

**Figure A2:**
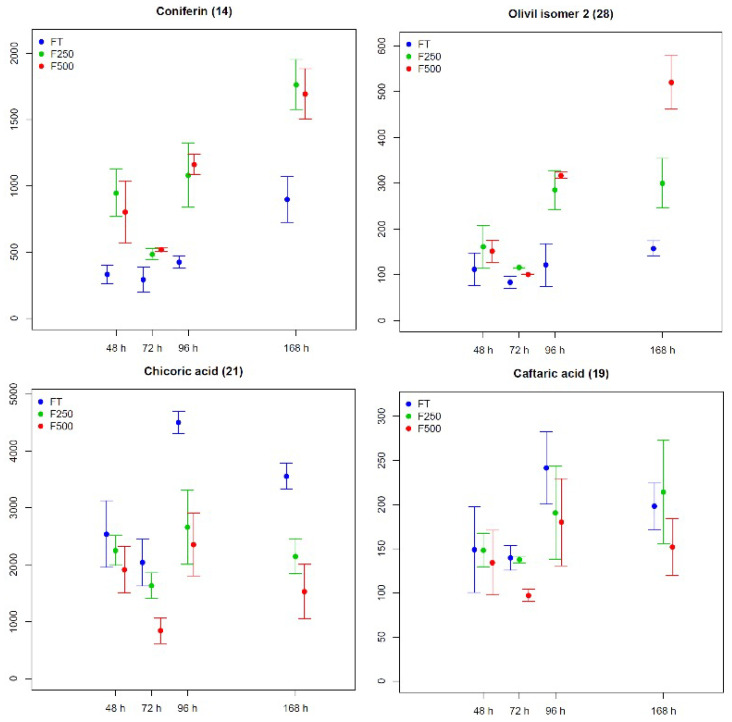
Comparison of some discriminating metabolite content in flax roots from control and seedling treated with COS oligosaccharides solutions (F250 and F500).

The relative levels of several discriminant metabolites responsible for the separation of the samples (shoots and roots) into clusters (control and COS treated seedlings) were examined in univariate analyses using a Kruskal and Wallis test (*p* < 0.05) (Figure A1 and Figure A2). Predefined metabolite profiling in shoots samples, chlorogenic acid (16), lotaustralin (2), linamarin (1), chaftoside isomer (13) and vicenin-2 (10) were investigated; the results are summarized in Figure A1. Interestingly, the biosynthesis of chlorogenic acid (16), lotaustralin (2) and linamarin (1) continued to increase gradually, reaching a maximum value at between 96 h and 168 h for control and COS treated seedlings with one or the other concentration. Compared to the control, this increase was more significant in the shoots of COS treated seedlings (i.e., it was dose-dependent) for chlorogenic acid (16); while it was less significant for lotaustralin (2) and linamarin (1).

In contrast, schaftoside isomer (13) and vicenin-2 (10) started to increase before 24 h, reaching a sharp peak at 48–72 h and thereafter gradually decreasing, ending with minimum values at 168 h for control and COS treated seedlings. This decrease was significantly more pronounced in samples of COS treated seedlings compared to the control. 

Some discriminant metabolites responsible for the separation of roots samples (Figure A2) into different groups corresponded to coniferin (14), olivil isomer 2 (28), olivil isomer 3 (29), chicoric acid (21) and caftaric acid (19). In the control and treated seedlings, the biosynthesis level of these metabolites increased over time to reach the maximum values at 168 h, except for caftaric acid (19) and chicoric acid (21), for which the maximum values were obtained at 96 h. Compared to the control, roots of COS treated seedlings (250 and 500 mg/L) exhibited significant dose-dependent increases in the biosynthesis of coniferin (14), olivil isomer 2 (28), olivil isomer 3 (29), while some doses showed the opposite effects on caftaric acid (19) and chicoric acid (21).

## 3. Discussion

Oligosaccharide signals obtained from chemical or enzymatic processes play an important role in the regulation of plant growth, development, defenses and interactions with other plants [60,61]. All these biological processes pass through distinct physiological stages and metabolic reprogramming, especially the regulation and accumulation of secondary metabolite levels in plants [62], which is often influenced by various environmental parameters [63]. 

In this context, COS oligosaccharides are considered to be potent elicitors of secondary metabolite accumulation in flax plants, although most elicitation experiments reported in the literature were conducted on suspension cells and callus culture [64,65,66]. To the best of our knowledge, to date, no study has been published on the use of COS oligosaccharides as elicitors in whole plant.

The seedling is the initial stage of growth for the flax, and one of the most sensitive periods to external factors [67]. It has been proposed that COS oligosaccharides first bind to specific receptors on root cell membranes; thus, they are involved in the modulation of growth and the plant’s defense system against biotic and abiotic stress [68,69].

COS oligosaccharides applied to plant using soil treatment could be readily adsorbed by natural colloids, and consequently, were unavailable for root adsorption. On the other hand, foliar spraying is strongly recommended in the agriculture and ecology fields because this treatment makes it possible to overcome certain obstacles related to soil properties and conditions including the leaching and immobilization of organic molecules like COS oligosaccharides. In addition, foliar applications of oligosaccharides elicitors can be implemented at opportune times in order to deliver biologically effective and uniform doses directly to the target organs [30]. In this study, a hydroponic experiment was applied as the in vivo model system to test the perception of COS oligosaccharides by the roots of flax seedlings [31]. Hydroponic systems provide a convenient means for studying plants in the laboratory while offering potential accessibility to all plant tissues. The impact of COS oligosaccharides on plants have often been evaluated by examining plant morphology and physiological and biochemical traits, including proteomic, metabolomic and transcriptomic data [16,20,33,34]. 

The influence of DP and degree of N-acetylation of COS oligosaccharides on their potential biological activities have been widely described in the literature [70,71,72]. Moreover, the existence of an optimal DP and DA of COS oligosaccharides was reported in previous studies; it depends on plant species and application modes [73,74]. Another important parameter to be considered is the concentration of COS oligosaccharides required in plant bioassays, which seems be associated with growth stage, cultivar or culture conditions. With regard of recently published articles on the elicitation of plants by oligosaccharides, the concentrations of COS applied in the culture medium vary over a broad range, from milligrams to grams per liter [75,76].

In the present study, we chose to work with two different concentration ranges of COS oligosaccharides (250 and 500 mg/L), thereby guaranteeing that the concentration of the average DP of the mixture was in the range investigated previously in the literature. 

According to our results, we found that the morphology and development of flax seedlings was significantly influenced in response to the presence of COS oligosaccharides. This was reinforced by observed symptoms (chlorosis in shoots, brown speckles in root), with visible signs including a decrease root length, shoot length and chlorophyll content. In this regard, there is systematic evidence in the literature that chitosan derivatives (chitin, chitosan, and oligosaccharides) are associated with root growth inhibition when applied at nonoptimal concentrations [77,78]. High doses of chitosan derivatives in the rhizosphere of some model plants were found to significantly arrest root growth [79,80]. The authors explained these results by assuming that chitosan derivatives caused strong alterations in the root cell morphology and cell division/elongation of treated seedlings.

As we have already mentioned, the presence of COS oligosaccharides in the hydroponic culture medium generated a biotic stress due to their characteristic of eliciting the defense mechanisms of plants against certain fungal pathogens. Likewise, they have been identified, isolated and characterized in numerous patho-systems models which are used to study plant-fungi interactions and are widely described in literature [30,68]. Moreover, COS oligosaccharides, when applied at high doses in a hydroponic medium, affect the nutritional status of the plant. The accumulation of macro- and micro- elements was altered in COS treated plants, exerting a negative impact on plant growth and development [31]. Significant change of these elements, essential for normal plant growth and development under the selected control conditions, generated another stress which was qualified as abiotic. The simultaneous accumulation of different stress factors triggered synergistic defense mechanisms in the flax seedlings in response to the situation. The responses of the plant appeared to depend on the severity of stress and its stage of growth. It can be postulated that seedling development was retarded or stopped by inhibiting the biosynthesis of growth substances, i.e., by redirecting its metabolism toward the production of defense molecules.

Plant stress responses consist of multiple spatio-temporally phases which are distinct from but are intimately related to ROS bursts [81]. At moderate concentrations, ROS have been conventionally considered as important mediators of intercellular communication and contribute to the signaling cascades of multiple plant responses.

Several studies have reported that basal ROS levels are positively correlated with stress levels, thus enhancing the susceptibility of cells to oxidative damage [82].

The two doses of COS oligosaccharides used in this study may have caused, directly or indirectly, stress which would induce ROS accumulation and alter the antioxidant systems in different parts of the roots. Moreover, the browning coloration of flax roots observed in the present study was attributed to cell membrane damage associated with the oxidation of phenolics. It was suggested that molecular oxygen works as a cosubstrate, leading to the production of *O*-quinones, which, once polymerized, results in the formation of brown pigments.

To maintain ROS homeostatic balance in cells, plants have developed sophisticated ROS-scavenging systems based on enzymatic and nonenzymatic antioxidant reactions [83]. When the plant experiences repetitive and severe stress, a potential additional mechanism is put in place based on a new class of biomolecules, i.e., so-called secondary metabolites, which complement the role of primary antioxidants in countering oxidative stress.

Some of these secondary metabolites are phenolic compounds that have been described in the literature as possessing antioxidant properties [84]. Therefore, they play an essential role in several physiological processes and are assumed to improve stress tolerance [85]. Most plants constitutively synthesize low concentrations of these phenolic compounds in both root and shoot tissues during normal growth and development [35,86].

Common examples of plant phenolic compounds are flavonoids, phenolic acids, and (neo)lignans. Each of these molecular families is represented by a diverse set of molecules whose chemical properties and concentrations depend largely on the species of plant and the target organ [19,21,64]. The list of these compounds can quickly become too large to enumerate. Therefore, the use of metabolomic analyses in this kind of study provides a selective advantage for the identification of the most discriminating compounds involved in the plant responses to biotic and abiotic stress [87].

As expected, we have shown in the context of this study that some of these secondary metabolites in flax seedlings (roots and shoots) are involved in the response to stress caused by the presence of COS in a hydroponic culture medium.

Thus, locally in the roots at the place of direct contact between COS and the flax seedlings, we observed a decrease in the chicoric acid content and an increase in (neo)lignans as well as coniferin, a precursor of lignin.

The data in this study showed an increase in coniferin content, a glucoside of monolignol coniferyl alcohol, in the roots treated by COS oligosaccharides. It was suggested that coniferin accumulation may be the result of both its increased biosynthesis and a decreased of conversion into other phenylpropanoid end products. In addition to being used in lignin and (neo)lignan formation [88], coniferin may be also utilized in other metabolic pathways which have not yet been characterized.

The increase in (neo)lignan content, produced by the roots following contact with COS, agrees with previously published data since (neo)lignans are considered as powerful antioxidants with the ability to neutralize highs levels of ROS [89].

Likewise, (neo)lignans are involved in the flax defense response against pathogens [90]. Moreover, other studies have putatively identified them in root exudates [91,92]. It is likely that root exudates act as mediators of mineral acquisition in low-nutrient environments and improve nutrient uptake.

The decrease in chicoric acid content in the roots is an astonishing result, because we expected an increase of this compound under stress conditions [16,93,94]. One explanation of this result is that chicoric acid is a principal antioxidant secondary metabolite mobilized into root cells to neutralize ROS [95]. During repetitive stress on flax roots caused by COS oligosaccharides, it may be that the balance between chicoric acid biosynthesis and its consumption as an antioxidant strongly favors the latter. On the other hand, in the case of multifactorial stress caused by COS oligosaccharides, flax seedlings tend to favor the production of defense-related molecules having the widest range of biological activities for the sake of saving energy and to cover a whole field of possibilities in terms of plant protection with the fewest possible molecules.

The systemic reaction of flax seedlings to the stresses generated by COS oligosaccharides applied locally to the roots was assessed at the shoot level. Shoots, being young organisms, attempt to protect against stress by mobilizing, among others, certain secondary metabolites involved in various defense mechanisms.

In this way, the putative accumulation of ROS in shoots cells could partly explain the increase of flavone *C*-glycosides of luteolin derivative contents in the shoots of COS-treated flax seedlings at the expense of apigenin *C*-glycosides derivatives. This result can be explained by the fact that luteolin *C*-glycoside derivatives possess higher antioxidant capacities than apigenin derivatives. Previous studies also noted similar shift from high accumulation of luteolin *C*-glycoside derivatives to lower levels of apigenin *C*-glycoside derivatives at distinct stages of flax seedling development [86]. The authors associated this stage of plant development with strong ROS accumulation under normal growth conditions.

In the same tissues, we observed a high induction of chlorogenic acid in COS-treated flax seedlings. Chlorogenic acid is a hydroxycinnamic acid synthesized by plants via the phenylpropanoid pathway. It is one of the most widely distributed soluble phenolic compounds in the plant kingdom. In vitro, this compound is reported to possess numerous biological activities including antioxidant properties and antibacterial activities [96,97]. It has been suggested that the antioxidant activities of chlorogenic acid may play an important role in the sustainable maintenance of plant health and the prevention of several diseases [20].

The application of COS oligosaccharides under the conditions applied in this study (high dose treatment with a prolonged exposure time) can be used to trigger metabolic response modification in planta, especially secondary metabolism. Obviously, before considering a field application of COS oligosaccharides on a flax crop, further studies are necessary to determine the effective dose to activate the synthesis and accumulation of plant chemical defenses (secondary metabolites) without negatively influencing plant development and growth.

## 4. Materials and Methods

### 4.1. Chitosan Oligosaccharides Preparation and Analysis

#### 4.1.1. Preparation of COS Oligosaccharides

The fungal chitosan solution (1 L) was prepared at 2% *w*/*v* concentration in 1% *v*/*v* acetic acid in a 2 L screw cap bottle. Hydrolysis of fungal chitosan was realized by autoclave treatment, in which one step corresponded to autoclaving the solution at 121 °C (1 bar) during 30 min with a 15 min temperature ramp-up. After autoclaving, the bottle was cooled in running tap water for 15 min. Various numbers of repeated cycles were investigated in this study.

The downstream process consisted of a first step of vacuum evaporation of the acetic acid, followed by adjustment of the pH to 8–9 with NaOH (1 M), allowing the nonhydrolyzed chitosan to be removed by centrifugation (2000× *g*, 20 min at 4 °C). The supernatant was then precipitated by 75% EtOH (7 *v*/*v*) and COS were collected by centrifugation (10,000× *g*, 20 min at 4 °C). The residue was washed in 75% EtOH in order to eliminate all traces of acetic acid. The final step consisted of solubilizing COS in distilled water, adjusting the pH to 6, and freeze-drying the supernatant obtained after centrifugation (10,000× *g*, 30 min at 4 °C). COS were obtained as a brownish powder. A HPLC/SEC analysis of COS oligosaccharides was performed according to the method described in a previously published study [38].

#### 4.1.2. Electrospray-Ionization High Resolution Mass Spectrometry (ESI-HRMS) Analysis

ESI-TOF HRMS experiments were performed using a SYNAPT G2-Si Q-TOF instrument hyphenated with the ACQUITY UPLC H-Class system (Waters, Manchester, UK). The mobile phase was composed of ammonium formate 50 mM and formic acid 0.1%, and the flow rate was set at 0.2 mL/min. One microliter of sample was injected, and the acquisition method run time was 2 min.

The ESI source was operating in positive ionization mode using a capillary voltage of 2.5 kV and the following conditions: cone voltage, 120 V; source offset, 20 V; source temperature, 120 °C; desolvation gas temperature, 450 °C; desolvation gas flow, 800 L/h, and cone gas flow, 50 L/h. Nitrogen (>99.5%) was employed as the desolvation gas. Mass calibration was carried out using a sodium formate solution, and lock mass correction was applied for accurate mass measurements using the [M−H] ions (*m*/*z* 554.2615) obtained from a Leu-enkephalin solution. The scan range was *m*/*z* 50–2500 at 0.25 s/scan. The TOF was operated in sensitivity mode, providing an average resolving power of 20,000 (FWHM). All spectra were recorded in continuum mode. Data acquisition was performed with MassLynx software (V4.1, Waters).

#### 4.1.3. NMR Analysis

The NMR (1D ^1^H, ^13^C) spectra were recorded in D_2_O (10 mg/mL) with TMSP as the internal standard at 25 °C on a Bruker Avance III 600 spectrometer (Bruker, Wissembourg, France). The spectrometer was operating at 600.13 MHz for ^1^H, using a three-nuclei (^1^H, ^13^C, ^15^N) inverse probe head TXI 5 mm z-gradient probe. Data acquisition and processing were performed using the TOPSPIN software (V3.6.2, Bruker).

### 4.2. Plant Material, Growth and COS Oligosaccharides Treatments

Flax seedlings cultivation was carried out according to a protocol described elsewhere [98].

Briefly, flax seeds were germinated on 1.1% agar medium at room temperature in the dark. Five-day-old seedlings were transferred to a hydroponic culture system on ½ Hoagland nutrient solutions [99,100]. Flax plants were grown for 4 weeks in a growth chamber (21 °C, 70% relative humidity, 120 µEinstein, 16 h/day). A total of 60 plants were obtained and divided into three groups, with a population grown on ½ Hoagland medium (control plants), two population grown on ½ Hoagland medium supplemented with different concentrations of COS oligosaccharides (250 and 500 mg/L). The treatment was performed in the middle of the photosynthetic period (after 8 h light). Flax shoot and root parts from each plant were harvested after COS oligosaccharide treatment and control at 48 h, 72 h, 96 h and 168 h respectively. For each dose and exposure time combination, five replicates were used. The pots were randomly chosen from each treatment to observe plant morphological parameters and analyze the metabolomic modifications in roots and shoots.

### 4.3. Metabolite Extractions

Metabolites from powdered roots and shoots (100 mg) were separately extracted using 500 µL of 1:1 water–methanol mixture. The samples were mixed with extraction solvent in a 1.5 mL Eppendorf centrifuge tube for 30 min at 60 °C using a ThermoMixer^®^ (Eppendorf AG, Hamburg, Germany) at 2000 rpm. The liquid phase was recovered by centrifugation (10 min, 10,000× *g*) and a total of 400 µL of the supernatant was collected. In order to achieve maximum metabolite extraction, this procedure was performed twice. The combined extracts were evaporated, and the dry residue was redissolved in 1 mL of a water–methanol mixture. The resulting extracts were analyzed by LC-MS method for the detection of various secondary metabolites.

### 4.4. Metabolite Analysis by LC/MS

#### 4.4.1. Samples Preparation

Extracts of shoots and roots were further diluted at an appropriate ratio with 1/1 methanol/water solvent. Subsequently, the diluted samples were filtered through 0.22 µm PTFE membrane and placed in glass vials for further LC-MS analysis. Quality control (QC) samples were prepared by pooling 10 µL from of each sample from the same plant part (roots, shoots). The QC samples were thoroughly mixed and analyzed regularly along with unknown samples. To reduce the effects of systematic errors, all samples were assigned to a random LC–MS run order and interspersed after every 20 injections with QC sample injections.

#### 4.4.2. UPLC-MS Data Acquisition

The UPLC–MS method was carried out following the same protocol and procedure described previously by Pontarin et al. [101]. UPLC-MS analysis was performed using a Waters ACQUITY UPLC I-Class system interfaced with a Vion IMS Q-TOF (Ion Mobility Quadrupole Time-of-flight) hybrid mass spectrometer, equipped with an electrospray ionization (ESI) source (Waters, Manchester, UK). The autosampler was programmed to inject 1 μL of each sample. Chromatographic separation of the analytes was carried out using a Kinetex Biphenyl (100 mm × 2.1 mm × 1.7 μm) column (Phenomenex), maintained at 55 °C.

The mobile phase was running at a flow rate of 0.55 mL/min with a run time of 10 min using the following gradient program (phase mobile A: water with formic acid 0.1%, phase mobile B: methanol with formic acid 0.1%): t = 0 min: 80:20, t = 0.5 min: 80:20, t = 5 min: 40:60, t = 6 min: 10:90, t = 7 min: 10:90, t = 7.5 min: 80:20, t = 10 min: 80:20.

The ESI parameters were set as follows: capillary voltage, 2.5 kV for negative mode; source temperature, 120 °C; desolvation temperature of 450 °C. Time-of-flight (TOF) MS was operated in sensitive mode. The data were acquired in high-definition MS^E^ (HDMS^E^) over a mass range of *m*/*z* 50–1200 at a mass resolving power of 50,000 FWHM and a scan time of 0.2 s.

#### 4.4.3. UPLC-MS Data Processing

Spectra were acquired and processed with the UNIFI software (version 1.9.4, Waters), allowing us to generate the data matrix with default parameters comprising the retention time, mass-to-charge ratio (*m*/*z*) values, and peak intensity. The untargeted metabolomics data matrix was cleaned by removing the variables with a significant variance (>35%) in the quality control (QC) and the variables present in the blank. A minimum intensity threshold was chosen to keep the variable. Metabolites were principally identified by matching the accurate masses, retention times and fragment pattern with those of the reference standards and literature references.

### 4.5. Data Analysis

A variance multiblock orthogonal partial least squares (AMOPLS) analysis was applied to assess the percentage of explained variance associated with each factor of the experimental design. The AMOPLS analysis was conducted after unit variance scaling as previously described [102,103] and computed using the MATLAB software (Matlab 2018, TheMathWorks, Natick, MA, USA). To evaluate significant differences (*p*-value < 0.05) between samples, according to Kruskall-Wallis tests, a multiple comparison was performed using the PMCMR (v. 4.3) and multcompView (v. 0.1–8) packages. Nonparametric Kruskall-Wallis tests were performed with the R software (version 4.0.3, company Foundation for Statistical Computing Vienna, Austria).

## 5. Conclusions

Plant growth and development may be affected positively or negatively by the use of oligosaccharides as elicitors. This study assessed the effects of COS oligosaccharides (DPs 2–11), obtained from chitosan fungal cell wall by the acidic/autoclaving hydrolysis method, on the growth and secondary metabolomics of flax seedlings.

COS oligosaccharides, applied at nonoptimal concentrations to the roots of flax seedlings during long-term treatment, triggered growth inhibition associated with the enhanced accumulation of (neo)lignans in the roots, as well as flavones luteolin *C*-glycosides and chlorogenic acid in the shoots. This is the first study to demonstrate induced phenolic compound accumulation in flax seedlings following treatment with COS oligosaccharides.

This study also provides new information on the chemical composition of flax seedlings by identifying olivils isomer (neo)lignans accumulation in the roots of COS-treated plants. The induced flavones *C*-glycosides and (neo)lignan compounds could be targeted as part of a large-scale purification strategy to obtain each compound individually for in vitro testing/confirmation of their antioxidant and antimicrobial roles *in planta*.

More importantly, such data provide information about the mechanisms put in place by flax plants, especially regarding the nature of the secondary metabolites involved in stress responses. Therefore, further investigations are required on the effect of COS oligosaccharides on flax seedlings under biotic and abiotic stress to determine the potential utility of COS in flax cultivation in sustainable agriculture. Indeed, it is necessary to take into account both ecological-environmental problems (water stress, cold stress for winter varieties, etc.) and biotic stress to comply with new standards, notably those concerning phytosanitary treatments. Thus, the long-term goal of this research is to maintain a significant level of farmer productivity in order to promote the cultivation of flax in the future.

## Figures and Tables

**Table 1:** Summary of metabolites identified and studied in the roots and shoots of flax seedlings.

Compound Number	Family	Compounds	Part Used	Identification
1	Cyanogenic glycoside	Linamarin	shoots, roots	annotation with standard
2	Cyanogenic glycoside	Lotaustralin	shoots, roots	annotation with standard
3	Flavonoids ^a^	Isoorientin	shoots	[22]
4	Flavonoids ^a^	Lucenin-2	shoots	[22]
5	Flavonoids ^a^	Carlinoside	shoots	[22]
6	Flavonoids ^a^	Carlinoside isomer	shoots	putative MS/MS annotation
7	Flavonoids ^b^	Isovitexin	shoots	[22]
8	Flavonoids ^b^	Vitexin	shoots	[22]
9	Flavonoids ^b^	Vicenin-1	shoots	[22]
10	Flavonoids ^b^	Vicenin-2	shoots	[22]
11	Flavonoids	Triticuside A	shoots	[22]
12	Flavonoids ^b^	Schaftoside	shoots	[22]
13	Flavonoids ^b^	Schaftoside isomer	shoots	putative MS/MS annotation
14	Hydroxycinnamic acids	Coniferin	roots	[22]
15	Hydroxycinnamic acids	HHMPG	roots	[22]
16	Hydroxycinnamic acids	3-*O*-caffeoylquinic acid	shoots	annotation with standard
17	Hydroxycinnamic acids	Caffeoylquinic acid isomer	shoots	putative MS/MS annotation
18	Hydroxycinnamic acids	Caffeoylquinic acid hexoside	shoots	putative MS/MS annotation
19	Hydroxycinnamic acids	Caftaric acid	roots	annotation with standard
20	Hydroxycinnamic acids	Icariside F2	roots	annotation with standard
21	Hydroxycinnamic acids	Chicoric acid	roots	annotation with standard
22	Lignans	PDG	shoots	[22]
23	Lignans	PMG	shoots	[22]
24	Lignans	DCG	shoots, roots	[22]
25	Lignans	LMG	shoots, roots	[22]
26	Lignans	(-)-Olivil 4′-*O*-beta-D-glucopyranoside	roots	annotation with standard
27	Lignans	Olivil isomer 1	roots	putative MS/MS annotation
28	Lignans	Olivil isomer 2	roots	putative MS/MS annotation
29	Lignans	Olivil isomer 3	roots	putative MS/MS annotation
30	Lignans	Olivil isomer 4	roots	putative MS/MS annotation
31	Lignans	SMG	roots	annotation with standard
32	Lignans	SMG isomer	roots	putative MS/MS annotation

^a^ Luteolin derivatives, ^b^ apigenin derivatives.

## Data Availability

The data presented in this study are available within the article, supplementary materials and Appendix A. Further information is available upon request from the corresponding author.

## Supplementary Materials

The following supporting information can be downloaded at: https://www.mdpi.com/article/10.3390/molecules27072372/s1, Figure S1: Electrospray-ionization mass spectrometry (ESI-MS) analysis of selected DP3 of chitosan oligosaccharides obtained from fungal chitosan by acetic acid hydrolyzed method using autoclaving-cooling process (10 cycles, 30 min at 121 °C and 15 min cooling); Table S1: Mass spectrometric data for COS oligomers produced during the acid hydrolysis process; Table S2: ^1^H-NMR and ^13^C-NMR spectral data for a solution of chitosan oligosaccharides solubilized in deuterium oxide (10 mg/mL); Table S3: Identification of phenolics compounds in roots and shoots of flax seedlings by UPLC-MS/MS; Table S4: Identification of phenolics compounds in roots and shoots of flax seedlings by UPLC-HDMS^E^.

## Sample Availability

Samples of the compounds are available on request from the corresponding author.
