# Supplementary material for: Secondary Metabolism Rearrangements in Linum usitatissimum L. after Biostimulation of Roots with COS Oligosaccharides from Fungal Cell Wall
Source: Molecules. 2022 Apr 6;27(7):2372. doi: 10.3390/molecules27072372 (PMC9000297; doi:10.3390/molecules27072372)
Supplement: Supplementary file 1 [file molecules-27-02372-s001.zip › molecules-1621567-supplementary.pdf]

SUPPLEMENTARY MATERIALS

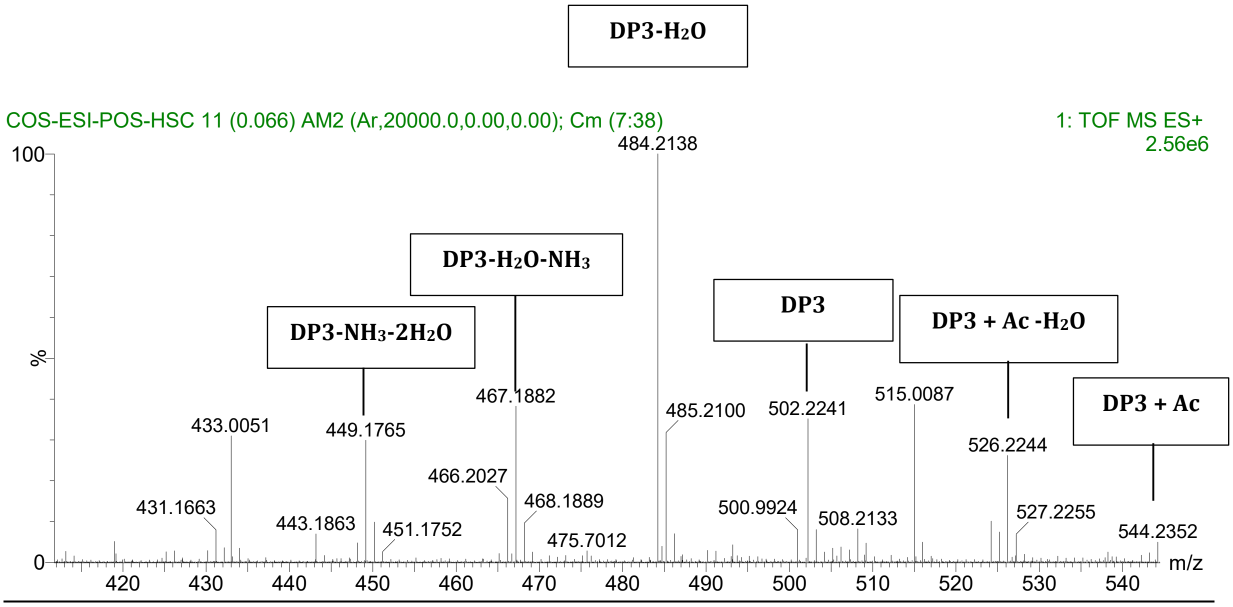

Figure S1. Electrospray-ionization high resolution mass spectrometry (ESI-HRMS) analysis of selected DP3 of chitosan oligosaccharides obtained from fungal chitosan by acetic acid hydrolyzed method using autoclaving-cooling process (10 cycles, 30 min at 121 °C and 15 min cooling).

Table S1: Mass spectrometric data for COS oligomers produced during the acid hydrolysis process.

	Elemental composition	Experimental m/z	Theoretical m/z	Error (ppm)	Error (mDa)
DP1-H_2O	$C_6H_{11}O_4N$	162.0766	162.0767	0.4	0.1
DP2-H_2O	$C_{12}H_{22}O_8N_2$	323.1454	323.1453	-0.4	-0.1
DP3-H_2O	$C_{18}H_{33}O_{12}N_3$	484.2143	484.2138	-0.9	-0.5
DP4-H_2O	$C_{24}H_{44}O_{16}N_4$	645.2831	645.2837	1.0	0.6
DP5-H_2O	$C_{30}H_{55}O_{20}N_5$	806.3519	806.3519	0.0	0.0
DP6-H_2O	$C_{36}H_{66}O_{24}N_6$	967.4207	967.4202	-0.5	-0.5
DP7- H_2O	$C_{42}H_{77}O_{28}N_7$	1128.4895	1128.4868	-2.4	-2.7
DP8-H_2O	$C_{48}H_{88}O_{32}N_8$	1289.5583	1289.5535	-3.7	-4.8
DP9-H_2O	$C_{54}H_{99}O_{36}N_9$	1450.6271	1450.621	-4.2	-6.1
DP10-H_2O	$C_{60}H_{110}O_{40}N_{10}$	1611.6959	1611.6892	-4.2	-6.7

Table S2: ^{1}H-NMR and ^{13}C-NMR spectral data for a solution of chitosan oligosaccharides solubilized in deuterium oxide (10 mg/mL).

Chemical shifts (δ) relative to TMSP *			
H_1	4.69	C_1	102.6
H_2	2.98	C_2	59.0
H_3	3.73	C_3	74.6
H_4	3.82	C_4	79.8
H_5	3.68	C_5	77.9
H_6	3.79	C_6	62.9
$H_{6'}$	3.95	-	-
C=O	-	C=O	177.4
CH_3	2.07	CH_3	24.9

* 3-(trimethylsilyl)-propionic acid D_4, sodium salt

Table S3: Identification of phenolics compounds in roots and shoots of flax seedlings by UPLC-MS/MS.

N	Family	Compounds	Part used	RT (min)	CCS (Å²)	[M-H]⁻ precursor ion (m/z)	MS² fragment ions (m/z)	m/z (Expected)	Error (mDa)	Error (ppm)	Elemental Composition	Collision energy (eV)	References
1	Cyanogenic glycoside	Linamarin	shoots, roots	0,54	165	292,1045		292,1038	0,7	2,2	$C_{11}H_{18}O_8N$*	16	[I, II]
							161,0454	161,0455	-0,2	-1,2	$C_6H_9O_5$		
							159,0304	159,0299	0,5	3,4	$C_6H_7O_5$		
							113,0240	113,0244	-0,4	-3,6	$C_5H_5O_3$		
							101,0240	101,0244	-0,4	-4,4	$C_4H_5O_3$		
							95,0133	95,0139	-0,5	-5,4	$C_5H_3O_2$		
							89,0239	89,0244	-0,5	-6,0	$C_3H_5O_3$		
							85,0289	85,0295	-0,6	-6,7	$C_4H_5O_2$		
							83,0137	83,0139	-0,2	-2,0	$C_4H_3O_2$		
							73,0289	73,0295	-0,6	-8,5	$C_3H_5O_2$		
							71,0133	71,0139	-0,5	-7,4	$C_3H_3O_2$		
2	Cyanogenic glycoside	Lotaustralin	shoots, roots	0,81	169	306,1204		306,1194	0,9	3,1	$C_{12}H_{20}O_8N$*	17	[I, II]
							161,0456	161,0455	0,0	0,0	$C_6H_9O_5$		
							159,0302	159,0299	0,3	1,8	$C_6H_7O_5$		
							113,0242	113,0244	-0,2	-1,7	$C_5H_5O_3$		
							101,0243	101,0244	-0,2	-1,6	$C_4H_5O_3$		
							99,0088	99,0088	0,0	0,2	$C_4H_3O_3$		
							95,0139	95,0139	0,0	0,0	$C_5H_3O_2$		
							89,0241	89,0244	-0,3	-3,1	$C_3H_5O_3$		
							85,0293	85,0295	-0,2	-2,2	$C_4H_5O_2$		
							73,0292	73,0295	-0,3	-3,9	$C_3H_5O_2$		
							71,0136	71,0139	-0,2	-3,4	$C_3H_3O_2$		
6	Flavonoids (luteolin derivatives)	*Carlinoside isomer*	shoots	2,44	230	579,1373		579,1355	1,8	3,0	$C_{26}H_{27}O_{15}$	32	[III]
							489,1059	489,1039	2,0	4,1	$C_{23}H_{21}O_{12}$		
							471,0947	471,0933	1,4	3,0	$C_{23}H_{19}O_{11}$		

							459,0948	459,0933	1,5	3,3	$C_{22}H_{19}O_{11}$		
							441,0835	441,0827	0,8	1,9	$C_{22}H_{17}O_{10}$		
							429,0845	429,0827	1,7	4,0	$C_{21}H_{17}O_{10}$		
							411,0741	411,0722	1,9	4,7	$C_{21}H_{15}O_{9}$		
							399,0729	399,0722	0,7	1,7	$C_{20}H_{15}O_{9}$		
							369,0630	369,0616	1,4	3,7	$C_{19}H_{13}O_{8}$		
							339,0514	339,0510	0,4	1,2	$C_{18}H_{11}O_{7}$		
13	Flavonoids (apigenin derivatives)	*Schaftoside isomer*	shoots	2,66	230	563,1426		563,1406	2,0	3,5	$C_{26}H_{27}O_{14}$	33	[III]
							503,1219	503,1195	2,4	4,8	$C_{24}H_{23}O_{12}$		
							473,1113	473,1089	2,3	4,9	$C_{23}H_{21}O_{11}$		
							443,1002	443,0984	1,9	4,2	$C_{22}H_{19}O_{10}$		
							425,0887	425,0878	0,9	2,0	$C_{22}H_{17}O_{9}$		
							413,0894	413,0878	1,6	3,8	$C_{21}H_{17}O_{9}$		
							383,0786	383,0772	1,4	3,7	$C_{20}H_{15}O_{8}$		
							353,0672	353,0667	0,5	1,4	$C_{19}H_{13}O_{7}$		
16	Hydroxycinnamic acids	3-O-caffeoylquinic acid	shoots	1,03	174	353,0885		353,0878	0,7	2,0	$C_{16}H_{17}O_{9}$	13	[IV, V]
							339,0514	339,0510	0,4	1,2	$C_{18}H_{11}O_{7}$		
							191,0561	191,0561	0,0	-0,2	$C_{7}H_{11}O_{6}$		
17	Hydroxycinnamic acids	*Caffeoylquinic acid isomer*	shoots	1,60	177	353,0887		353,0878	0,9	2,6	$C_{16}H_{17}O_{9}$	13	[IV, V]
							191,0561	191,0561	0,0	-0,1	$C_{7}H_{11}O_{6}$		
18	Hydroxycinnamic acids	*Caffeoylquinic acid hexoside*	shoots	0,62	231	515,1423		515,1406	1,6	3,2	$C_{22}H_{27}O_{14}$	25	[IV, V]
							353,0891	353,0878	1,3	3,6	$C_{16}H_{17}O_{9}$		
							191,0562	191,0561	0,1	0,6	$C_{7}H_{11}O_{6}$		
19	Hydroxycinnamic acids	Caftaric acid	roots	0,68	172	333,0224		333,0252	-2,8	-8,4	$C_{15}H_{9}O_{9}$	14	[V]
20	Hydroxycinnamic acids	Icariside F2	roots	1,78	204	447,1520		447,1508	1,2	2,8	$C_{19}H_{27}O_{12}$*	10	[VI, VII, VIII]
							402,1498	402,1531	-3,4	-8,3	$C_{18}H_{26}O_{10}$		
							401,1463	401,1453	0,9	2,3	$C_{18}H_{25}O_{10}$		
21	Hydroxycinnamic	Chicoric acid	roots	2,77	204	473,0739		473,0726	1,4	2,9	$C_{22}H_{17}O_{12}$	13	[V]

	acids						311,0414	311,0409	0,5	1,7	$C_{13}H_{11}O_9$		
							293,0303	293,0303	0,0	0,1	$C_{13}H_9O_8$		
							179,0347	179,0350	-0,2	-1,4	$C_9H_7O_4$		
							149,0088	149,0092	-0,4	-2,6	$C_4H_5O_6$		
26	Lignans	(-)-Olivil 4'-O-beta-D-glucopyranoside	roots	2,57	231	583,2041		583,2032	0,8	1,4	$C_{27}H_{35}O_{14}$*	12	[IX, X, XI, XII, XIII]
							538,2019	538,2056	-3,7	-6,9	$C_{26}H_{34}O_{12}$		
							537,1984	537,1978	0,7	1,3	$C_{26}H_{33}O_{12}$		
							375,1453	375,1449	0,3	0,9	$C_{20}H_{23}O_7$		
27	Lignans	*Olivil isomer 1*	roots	1,64	238	583,2052		583,2032	2,0	3,4	$C_{27}H_{35}O_{14}$	19	[IX, X, XI, XII, XIII]
							507,1873	507,1872	0,1	0,3	$C_{25}H_{31}O_{11}$		
							375,1459	375,1449	0,9	2,5	$C_{20}H_{23}O_7$		
							357,1348	357,1344	0,4	1,3	$C_{20}H_{21}O_6$		
							345,1347	345,1344	0,3	0,9	$C_{19}H_{21}O_6$		
							315,1240	315,1238	0,2	0,8	$C_{18}H_{19}O_5$		
							297,1139	297,1132	0,7	2,2	$C_{18}H_{17}O_4$		
28	Lignans	*Olivil isomer 2*	roots	2,63	223	583,2042		583,2032	1,0	1,6	$C_{27}H_{35}O_{14}$	17	[IX, X, XI, XII, XIII]
							537,1998	537,1978	2,0	3,7	$C_{26}H_{33}O_{12}$		
							375,1460	375,1449	1,0	2,7	$C_{20}H_{23}O_7$		
							327,1248	327,1238	1,0	3,1	$C_{19}H_{19}O_5$		
							195,0663	195,0663	0,0	0,0	$C_{10}H_{11}O_4$		
29	Lignans	*Olivil isomer 3*	roots	2,90	222	583,2050		583,2032	1,8	3,1	$C_{27}H_{35}O_{14}$	17	[IX, X, XI, XII, XIII]
							537,1990	537,1978	1,2	2,3	$C_{26}H_{33}O_{12}$		
							375,1457	375,1449	0,8	2,1	$C_{20}H_{23}O_7$		
							327,1244	327,1238	0,6	1,7	$C_{19}H_{19}O_5$		
							195,0661	195,0663	-0,2	-1,1	$C_{10}H_{11}O_4$		
							179,0713	179,0714	-0,1	-0,4	$C_{10}H_{11}O_3$		
							165,0555	165,0557	-0,2	-1,1	$C_9H_9O_3$		

30	Lignans	*Olivil isomer 4*	roots	3,29	237	583,2019		583,2032	-1,3	-2,3	$C_{27}H_{35}O_{14}$	17	[XIV, XV]
32	Lignans	*SMG isomer*	roots	3,90	217	523,2191		523,2185	0,6	1,1	$C_{26}H_{35}O_{11}$	15	[XIV,XV]
							361,1662	361,1657	0,5	1,4	$C_{20}H_{25}O_{6}$		

* [M+HCOO]- adduct

Table S4: Identification of phenolics compounds in roots and shoots of flax seedlings by UPLC- HDMS^E.

N	Family	Compounds	Part used	RT (min)	CCS (Å^2)	[M-H]$^-$ precursor ion (m/z)	MS^E fragment ions (m/z)	m/z (Expected)	Error (mDa)	Error (ppm)	Elemental Composition	References
1	Cyanogenic glycoside	Linamarin	shoots, roots	0,54	165	292,1035		292,1038	-0,3	-1,0	$C_{11}H_{18}O_8N$*	[I, II]
							246,0979	246,0983	-0,4	-1,6	$C_{10}H_{16}O_6N$	
2	Cyanogenic glycoside	Lotaustralin	shoots, roots	0,81	169	306,1189		306,1194	-0,5	-1,6	$C_{12}H_{20}O_8N$*	[I, II]
							260,1133	260,114	-0,7	-2,7	$C_{11}H_{18}O_6N$	
6	Flavonoids (luteolin derivatives)	*Carlinoside isomer*	shoots	2,44	230	579,1362		579,1362	0	0,0	$C_{26}H_{27}O_{15}$	[III]
							489,1046	489,1038	0,8	1,6	$C_{23}H_{21}O_{12}$	
							459,0942	459,0927	1,5	3,3	$C_{22}H_{19}O_{11}$	
							429,0836	429,0827	0,9	2,1	$C_{21}H_{17}O_{10}$	
							399,0731	399,0722	0,9	2,3	$C_{20}H_{15}O_9$	
							369,0619	369,0616	0,3	0,8	$C_{19}H_{13}O_8$	
							339,0510	339,051	0	0,0	$C_{18}H_{11}O_7$	
13	Flavonoids (apigenin derivatives)	*Schaftoside isomer*	shoots	2,66	231	563,1403		563,1406	-0,3	-0,5	$C_{26}H_{27}O_{14}$	[III]
							503,1206	503,1195	1,1	2,2	$C_{24}H_{23}O_{12}$	
							443,0982	443,0984	-0,2	-0,5	$C_{22}H_{19}O_{10}$	
							383,0767	383,0772	-0,5	-1,3	$C_{20}H_{15}O_8$	
16	Hydroxycinnamic acids	3-O-caffeoylquinic acid	shoots	1,03	174	375,0697		375,0692	0,5	1,3	$C_{16}H_{16}O_9Na$**	[IV, V]
							353,0880	353,0878	0,2	0,6	$C_{16}H_{17}O_9$	
							191,0558	191,0561	-0,3	-1,6	$C_7H_{11}O_6$	
							135,0448	135,0452	-0,4	-3,0	$C_8H_7O_2$	
							134,0366	134,0373	-0,7	-5,2	$C_8H_6O_2$	
							133,0291	133,0295	-0,4	-3,0	$C_8H_5O_2$	
17	Hydroxycinnamic acids	*Caffeoylquinic acid isomer*	shoots	1,6	177	375,0697		375,0692	0,4	1,1	$C_{16}H_{16}O_9Na$**	[IV, V]
							353,0881	353,0878	0,3	0,8	$C_{16}H_{17}O_9$	
							191,0561	191,0561	0	0,0	$C_7H_{11}O_6$	

18	Hydroxycinnamic acids	*Caffeoylquinic acid hexoside*	shoots	0,62	231	537,1230		537,122	1	1,9	$C_{22}H_{26}O_{14}Na$**	[IV, V]
							515,1403	515,1403	0	0,0	$C_{22}H_{27}O_{14}$	
19	Hydroxycinnamic acids	Caftaric acid	roots	0,68	172	333,0224		333,0228	-0,4	-1,2	$C_{13}H_{10}O_9Na$**	[V]
							311,0404	311,0409	-0,5	-1,6	$C_{13}H_{11}O_9$	
							135,0449	135,0452	-0,3	-2,2	$C_8H_7O_2$	
							134,0371	134,0373	-0,2	-1,5	$C_8H_6O_2$	
20	Hydroxycinnamic acids	Icariside F2	roots	1,78	204	447,1512		447,1508	0,4	0,9	$C_{19}H_{27}O_{12}$*	[VI, VII, VIII]
							401,1458	401,1453	0,5	1,2	$C_{18}H_{25}O_{10}$	
21	Hydroxycinnamic acids	Chicoric acid	roots	2,77	204	495,0550		495,054	1	2,0	$C_{22}H_{16}O_{12}Na$**	[V]
							473,0734	473,0725	0,9	1,9	$C_{22}H_{17}O_{12}$	
							311,0408	311,0403	0,5	1,6	$C_{13}H_{11}O_9$	
							293,0303	293,0297	0,6	2,0	$C_{13}H_9O_8$	
							219,0297	219,0299	-0,2	-0,9	$C_{11}H_7O_5$	
							179,0346	179,035	-0,4	-2,2	$C_9H_7O_4$	
							149,0089	149,0092	-0,3	-2,0	$C_4H_5O_6$	
							135,0446	135,0452	-0,6	-4,4	$C_8H_7O_2$	
							134,0367	134,0373	-0,6	-4,5	$C_8H_6O_2$	
26	Lignans	(-)-Olivil 4'-O-beta-D-glucopyranoside	roots	2,57	231	583,2041		583,2032	0,9	1,5	$C_{27}H_{35}O_{14}$*	[IX, X, XI, XII, XIII]
							537,1988	537,1978	1	1,9	$C_{26}H_{33}O_{12}$	
							375,1456	375,1449	0,7	1,9	$C_{20}H_{23}O_7$	
27	Lignans	*Olivil isomer 1*	roots	1,64	238	583,2040		583,2032	0,8	1,4	$C_{27}H_{35}O_{14}$	[IX, X, XI, XII, XIII]
							537,1960	537,1978	-1,8	-3,4	$C_{26}H_{33}O_{12}$	
							297,1129	297,1132	-0,3	-1,0	$C_{18}H_{17}O_4$	
28	Lignans	*Olivil isomer 2*	roots	2,63	223	583,2032		583,2032	0	0,0	$C_{27}H_{35}O_{14}$	[IX, X, XI, XII, XIII]
							537,1972	537,1978	-0,6	-1,1	$C_{26}H_{33}O_{12}$	
29	Lignans	*Olivil isomer 3*	roots	2,9	222	583,2029		583,2032	-0,3	-0,5	$C_{27}H_{35}O_{14}$	[IX, X, XI, XII, XIII]
							537,1966	537,1978	-1,2	-2,2	$C_{26}H_{33}O_{12}$	

30	Lignans	*Olivil isomer 4*	roots	3,29	237	583,2019		583,2032	-1,3	-2,2	$C_{27}H_{35}O_{14}$	[IX, X, XI, XII, XIII]
31	Lignans	SMG	roots	4,35	225	523,2185		523,2185	0	0,0	$C_{26}H_{35}O_{11}$	[XIV, XV]
					217	523,2189		523,2185	0,4	0,8	$C_{26}H_{35}O_{11}$	
32	Lignans	*SMG isomer*	roots	3,9			361,1651	361,1657	-0,6	-1,7	$C_{20}H_{25}O_{6}$	[XIV, XV]
							346,1410	346,1422	-1,2	-3,5	$C_{19}H_{22}O_{6}$	

* [M+HCOO]- adduct

** [M-2H+Na]- adduct

References

1. Sulyok, M.; Beed, F.; Boni, S.; Abass, A.; Mukunzi, A.; Krska, R. Quantitation of multiple mycotoxins and cyanogenic glucosides in cassava samples from Tanzania and Rwanda by an LC-MS/MS-based multi-toxin method. *Food Additives & Contaminants: Part A*. **2014**, 32(4), 488-502, doi.org/10.1080/19440049.2014.975752.
2. Dalisay, D; Kim, K; Lee, C; Yang, H; Rübel, O; Bowen, B; Davin, L; Lewis, N. Dirigent Protein-Mediated Lignan and Cyanogenic Glucoside Formation in Flax Seed: Integrated Omics and MALDI Mass Spectrometry Imaging. *Journal of Natural Products*. **2015**, *78*, 1231-1242, doi: 10.1021/acs.jnatprod.5b00023.
3. Tchoumtchoua, J.; Mathiron, D.; Pontarin, N.; Gagneul, D.; van Bohemen, A.-I.; Otogo N'nang, E.; Mesnard, F.; Petit, E.; Fontaine, J.-X.; Molinié, R.; et al. Phenolic Profiling of Flax Highlights Contrasting Patterns in Winter and Spring Varieties. Molecules **2019**, 24, 4303:1-4303 :14, doi:10.3390/molecules24234303.
4. Bujor, O. Extraction, identification and antioxidant activity of the phenolic secondary metabolites isolated from the leaves, stems and fruits of two shrubs of the Ericaceae family. *PhD Thesis*. **2016**.
5. Chen, C; Chen, S; Liu, C; Wu, D; Kuo, C; Lin, C; Chou, H; Wang, Y; Tsai, Y; Lai, M; Chung, C. Invasion and Colonization Pattern of *Fusarium fujikuroi* in Rice. *Fungal Biology and Genetics*. **2020**, *110*, 1934-1945, doi: 10.1094/PHYTO-03-20-0068-R.
6. Wang, M; Li, J; Rangarajan, M; Shao, Y; LaVoie, E; Huang, T; Ho, C. Antioxidative Phenolic Compounds from Sage (*Salvia officinalis*). *Journal of Agricultural and Food Chemistry*. **1998**, *46*, 4869-4873, doi: 10.1021/jf980614b.
7. Xiao, S; Hao, C; Ai, N; Luo, K; Wen, X; Wang, S; Fan, X. Deciphering the differentiations of traditional Chinese medicine analogous formulae by parallel liquid chromatography-mass spectrometry coupled with microplate-based assays. *Analytical Methods*. **2014**, *6*, 9283, doi: 10.1039/c4ay01972e.
8. Bai, M; Shi, W; Tian, J; Lei, M; Kim, Y; Sun, Y; Kim, J; Gao, J. Soluble epoxide hydrolase inhibitory and anti-inflammatory components from the leaves of *Eucommia ulmoides* Oliver (Duzhong). *Journal of Agricultural and Food Chemistry*. **2015**, *63*, 2198-2205, doi: 10.1021/acs.jafc.5b00055.
9. Schumacher, B; Scholle, S; Hölzl, J; Khudeir, N; Hess, S; Müller, C. Lignans Isolated from Valerian: Identification and Characterization of a New Olivil Derivative with Partial Agonistic Activity at A1 Adenosine Receptors. *Journal of Natural Products*. **2002**, *65*, 1479-1485, doi: 10.1021/np010464q.
10. Kanchanapoom, T; Noiarsa, P; Otsuka, H ; Ruchirawat, S. Lignan, phenolic and iridoid glycosides from *Stereospermum cylindricum*. *Phytochemistry*. **2006**, *67*, 516-520, doi: 10.1016/j.phytochem.2005.10.009.
11. Deyama, T; Ikawa, T; Kitagawa, S ; Nishibe, S. The Constituents of Eucomia ulmoides OLIV. III. Isolation and Structure of a New Lignan Glycoside. *Chemical and Pharmaceutical Bulletin*. **1986**, *34*(2), 523-527, doi: 10.1248/cpb.34.523.
12. Kadowaki, E; Yoshida, Y; Nitoda, T; Baba, N; Nakajima, S. (-)-Olivil and (+)-1-Acetoxypinoresinol from the Olive Tree (*Olea europaea* LINNE; Oleaceae) as Feeding Stimulants of the Olive Weevil (*Dyscerus perforates*). *Bioscience, Biotechnology, and Biochemistry*. **2003**, *67*(2), 415-419, doi: 10.1271/bbb.67.415.
13. Shi, S; Peng, M; Zhang, Y; Peng, S. Combination of preparative HPLC and HSCCC methods to separate phosphodiesterase inhibitors from *Eucommia ulmoides* bark guided by ultrafiltration-based ligand screening. *Analytical and Bioanalytical Chemistry*. **2013**, 405:4213-4223, doi: 10.1007/s00216-013-6806-4.
14. Ghose, K; Selvaraj, K; McCallum, J; Kirby, C; Sweeney-Nixon, M; Cloutier, S; Deyholos, M; Datla, R; Fofana, B. Identification and functional characterization of a flax UDP-glycosyltransferase glucosylating secoisolariciresinol (SECO) into secoisolariciresinol monoglucoside (SMG) and diglucoside (SDG). *BMC Plant Biology*. **2014**, 14:82, doi: 10.1186/1471-2229-14-82.
15. Huis, R; Morreel, K; Fliniaux, O; Lucau-Danila, A; Fénart, S; Grec, S; Neutelings, G; Chabbert, B; Mesnard, F; Boerjan, W; Hawkins, S. Natural Hypolignification Is Associated with Extensive Oligolignol Accumulation in Flax Stems. *Plant Physiology*. **2012**, *158*, 1893-1915, doi: 10.1104/pp.111.192328.
